# Trends in the incidence and mortality of legionellosis in Japan: a nationwide observational study, 1999–2017

**DOI:** 10.1038/s41598-021-86431-8

**Published:** 2021-03-31

**Authors:** Shinnosuke Fukushima, Hideharu Hagiya, Yuki Otsuka, Toshihiro Koyama, Fumio Otsuka

**Affiliations:** 1 Department of General Medicine, Graduate School of Medicine, Dentistry, and Pharmaceutical Sciences, Okayama University, 2-5-1 Shikata-cho, Kitaku, Okayama 700-8558 Japan; 2 Department of Pharmaceutical Biomedicine, Graduate School of Medicine, Dentistry, and Pharmaceutical Sciences, Okayama University, 1-1-1 Tsushima-Naka, Kita-ku, Okayama, 700-8530 Japan

**Keywords:** Microbiology, Climate sciences, Diseases, Medical research

## Abstract

This study examined temporal trend, seasonality, and geographical variations of legionellosis incidence and mortality in Japan. This nationwide observational study used the Japanese Vital Statistics and Infectious Diseases Weekly Report (1999–2017) data to calculate legionellosis crude and age-adjusted incidence and mortality rates per 100,000 population by age and sex. Incidence was compared among the 4 seasons and regional incidence among 47 prefectures. Of 13,613 (11,194 men) people with legionellosis in Japan, 725 (569 men) were fatal. Increasing incidence trend occurred from 0.0004 (1999) to 1.37 (2017) per 100,000 population. People aged ≥ 70 years accounted for 43.1% overall; men’s age-adjusted incidence rate was consistently approximately five times higher than for women. Significantly higher incidence occurred in summer than in winter (*p* = 0.013). Geographically, highest incidence (≥ 2.0 per 100,000 population) occurred in Hokuriku District, with increasing trends in Hokkaido and middle-part of Japan. Estimated fatality rates decreased consistently at 5.9% (95% confidence interval: − 8.1, − 3.5) annually, from 1999 to 2017, with no trend change point. Increasing legionellosis incidence occurred in Japan during 1999–2017, with declining estimated fatality rates. In this aging society and warming world, disease clinical burden may further deteriorate in future due to increasing incidence trends.

## Introduction

Legionellosis, clinically classified into Legionella pneumonia (Legionnaires’ disease) and Pontiac fever, is caused by *Legionella* species, which grow in free-living, ubiquitous, freshwater and soil amoebae^1^. The bacteria also contaminate artificial water systems, such as cooling towers, hot and cold water systems, and whirlpool spas, from where humans contract the disease via inhalation of contaminated aerosols^2^. Legionella pneumonia has been known to cause severe respiratory disease requiring hospitalisation, potentially yielding higher fatality. Pontiac fever generally presents as a flu-like illness that resolves spontaneously.

Since the first clinical isolation of the organism from humans in the United States in 1977^3^, growing numbers of legionellosis has been documented worldwide through surveillance systems to track the disease across North America, New Zealand, Australia, Europe, and other countries^4–9^. In Ontario, for example, the overall incidence rate of legionellosis increased from 0.1 to 2.0 cases per 100,000 population between 2004 and 2013^6^. By sex, cases were predominant in males (0.55 per 100,000) compared to females (0.35 per 100,000). Approximately three quarters of the cases were aged ≥ 50 years, with a strong linear trend between aging and incidence of the disease; incidence rate ratio per decade age increase is 1.67 rate per 100,000 population^6^.

In addition to the increasing trends, the seasonality of legionellosis incidence has been documented worldwide. For example, a rise occurred in the number of notification of the disease from June–October in the United States^9^, July–August in Canada^10^, and August–November in Europe^4^. In the southern hemisphere, in New Zealand for instance, its incidence increases between the late spring (September–November) and autumn (March–May)^5^.

Effects of climate and environmental changes on the onset of legionellosis are gradually being reported. According to a previous study in Italy, the increasing risk of legionellosis is associated with increases in precipitation and average temperature^11^. Decreases in daily temperature excursion (difference between maximum and minimum temperature over the course of 24 h during the incubation period of disease) and minimum temperature are also related with the rise^11^. Climate change potentially leads to increased seawater temperature, drought, higher rainfall, rising sea levels, and flooding, which could play an important role in the transmission of waterborne diseases, including legionellosis^12, 13^.

Case fatality of the disease also matters. In Europe, of 23,164 cases of the disease that were investigated between 2011 and 2015, 2,161 (9.3%) died, although the case fatality ratio decreased from 10.5% to 8.1%^14^. In Canada, 84% of legionellosis cases were hospitalised, with a fatality rate of 9.5% 6. In the United States, among 5,503 cases of legionellosis with a known outcome, 456 (8.3%) died^15^. These data suggest the severity of the disease that is commonly observed worldwide.

Based on public reports by the National Institute of Infectious Diseases (NIID) of Japan, the number of notifications of legionellosis has also been increasing over time. However, to our knowledge, there are no published reports on the long-term trends in the incidence and mortality of legionellosis in Japan that consider seasonal and geographical variation. Our objectives for this study were to examine the temporal trend, seasonality, and geographical variations in the incidence of legionellosis in Japan over the past nearly 20 years. Also, we estimated the case-fatality rate of the disease in Japan, with its chronological trend. Analysing the recent trends in legionellosis incidence in Japan will inform the epidemiological characteristics at the global level and provide additional data for better public health control measures.

## Results

### The reported numbers, crude, and age-adjusted rates (AARs) of legionellosis incidence

**Figure 1:**
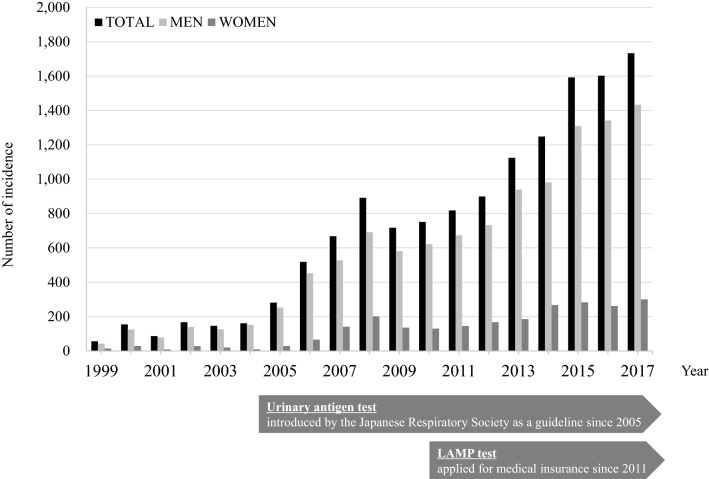
Chronological changes in the number of reported cases of legionellosis in Japan, 1999–2017. The urinary antigen test was introduced in a clinical guideline by the Japanese Respiratory Society in 2005. Then, LAMP (Loop-mediated Isothermal Amplification) method for the diagnosis of legionellosis was approved by the medical insurance in 2011.

**Figure 2:**
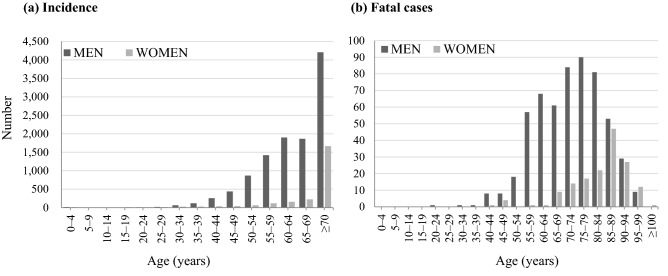
Age-sex distribution of the incidence **(a)** and number of fatal cases **(b)** of legionellosis in Japan, 1999–2017.

During the study period, 13,613 individuals (11,194 men and 2419 women) developed legionellosis in Japan. The incidence of legionellosis rose from 1999 to 2017 (1733 cases overall; with 1433 in men and 300 in women) (Fig. 1). Distribution of the incidence by age and sex during 1999–2017 is depicted in Fig. 2a. The incidences increased with age in both sexes, and those aged ≥ 70 years accounted for 43.1% of the total number of cases. Crude rate and AAR of the incidence per 100,000 population increased, reaching up to 1.37 and 1.05 in 2017, respectively (Table 1). Between sexes, AAR of the incidence among men has remained approximately 5 times higher than that in women, between 1999 and 2017. Overall, the annual incidence rate increased at a rate of 32.3% from 1999–2007, and then slowly but continuously rose by 11.1% from 2007–2017. The average annual percentage change (AAPC) of the incidence rate was 20.1% (95% confidence interval [95% CI]; 14.7 to 25.7) during the entire study period. By sex, annual percentage change (APC) among men increased continuously with an AAPC of 19.8% (95% CI; 14.9 to 24.9). While among women, the APC increased by 8.3% between 2007 and 2017, but the AAPC did not show a significant change (Table 2).

**Table 1:** Numbers, crude rates (CR), and age-adjusted rates (AAR) of incidence and mortality of legionellosis per 100,000 population in Japan, 1999–2017.

Year	Incidence					Mortality		
	Case no. (total)	CR (total)	AAR (total)	AAR (men)	AAR (women)	Case no. (total)	CR (total)	AAR (total)
1999	56	0.04	0.04	0.07	0.02	11	0.01	0.01
2000	154	0.12	0.12	0.20	0.05	12	0.01	0.01
2001	86	0.07	0.06	0.12	0.01	13	0.01	0.01
2002	139	0.13	0.13	0.21	0.04	15	0.01	0.01
2003	146	0.11	0.11	0.19	0.03	14	0.01	0.01
2004	161	0.13	0.12	0.22	0.01	8	0.01	0.01
2005	211	0.22	0.20	0.36	0.04	21	0.02	0.01
2006	518	0.41	0.36	0.64	0.09	31	0.02	0.02
2007	668	0.52	0.45	0.73	0.19	31	0.02	0.02
2008	892	0.70	0.60	0.94	0.26	46	0.04	0.03
2009	717	0.56	0.47	0.78	0.18	58	0.05	0.03
2010	751	0.59	0.49	0.83	0.17	43	0.03	0.03
2011	818	0.64	0.53	0.88	0.18	56	0.04	0.03
2012	899	0.71	0.57	0.95	0.20	58	0.05	0.03
2013	1124	0.88	0.72	1.23	0.21	64	0.05	0.03
2014	1248	0.98	0.76	1.23	0.30	65	0.05	0.03
2015	1592	1.25	0.98	1.66	0.32	59	0.05	0.03
2016	1602	1.26	0.98	1.67	0.29	67	0.05	0.03
2017	1733	1.37	1.05	1.77	0.33	53	0.04	0.03

**Table 2:** Trend analysis in crude rates of the incidence and mortality of legionellosis per 100,000 population by sex, 1999–2017.

	Period 1		Period 2		Period 3		Entire study period
	Years	APC (95% CI)	Years	APC (%)	Years	APC (%)	Average APC (95% CI)
**Incidence**							
All	1999–2007	32.3 (19.0 to 47.0)	2007–2017	11.1 (7.8 to 14.5)			20.1 (14.7 to 25.7)
Men	1999–2007	31.1 (19.1 to 44.4)	2007–2017	11.4 (8.3 to 14.6)			19.8 (14.9 to 24.9)
Women	1999–2004	−7.3 (−38.8 to 40.5)	2004–2007	101.5 (−44.1 to 626.7)	2007–2017	8.3 (3.7 to 13.1)	15.0 (−7.4 to 43)
**Mortality**							
All	1999–2017	−5.9 (−8.1 to −3.5)					−5.9 (−8.1 to −3.5)
Men	1999–2007	−13.1 (−21.6 to −3.6)	2007–2011	11.5 (−17.8 to 51.3)	2011–2017	−14.5 (−21.9 to −6.5)	−8.6 (−15.5 to −1.3)
Women	1999–2017	−5.2 (−8.0 to −2.2)					−5.2 (−8.0 to −2.2)

*APC* annual percentage change, *CI* confidence interval.

### Seasonality of the incidence

**Figure 3:**
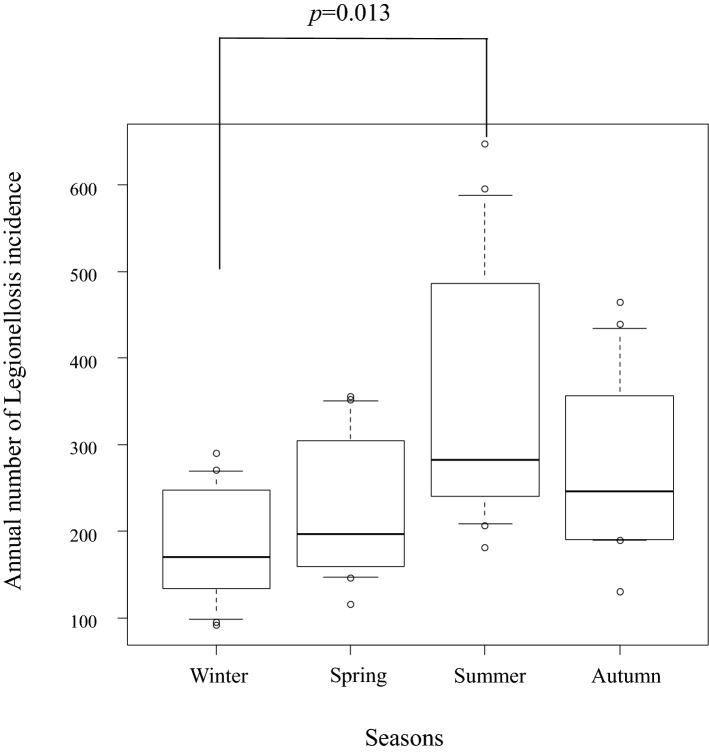
Comparison of the incidence of legionellosis among the 4 seasons, 2006–2017. Shown are the box and whisker plots with median and 10th to 90th percentiles. Mann–Whitney U test revealed a significant difference (p = 0.013) between winter and summer.

Data between 2006 and 2017 (12 years) was applied for the seasonality comparison. Median numbers (interquartile ranges) of legionellosis incidence in winter, spring, summer, and autumn were 170 (135.25, 241); 197 (162, 289.5); 282.5 (241.75, 467.5); and 246.5 (190.75, 340), respectively. The Kruskal–Wallis test was used to calculate the P-value of < 0.001 among the 4 seasons, and Mann–Whitney U test with Bonferroni adjustment demonstrated a significant difference between winter and summer (*p* = 0.013) (Fig. 3).

### Geographic distribution

**Figure 4:**
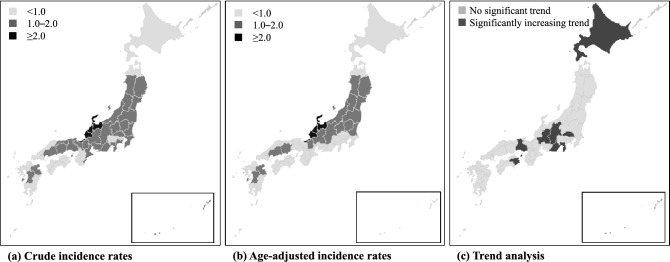
Incidence rates of legionellosis **(a)** crude incidence rates; **(b)** age-adjusted incidence rates; **(c)** trend analysis, 2013–2017. In the trend analysis, we calculated the annual percentage change in each of the 47 prefectures by joinpoint analysis.

We utilised the regional data (each of 47 prefectures in Japan) between 2013 and 2017 (the last 5 years). 2 maps (Fig. 4a,b) showing differences of the crude- and age-adjusted incidence rates of legionellosis. Data in detail are as summarised in the table (Supplementary Table S1). The incidence rates were highest in 3 prefectures in the Hokuriku District, with ≥ 2.0 per 100,000 population. Toyama Prefecture had the highest reported annual incidence rate of 3.49 per 100,000 population, followed by Ishikawa (2.81) and Fukui (2.04). Otherwise, no notable regional biases were observed. Regional increasing trends were examined by the Joinpoint analysis, which showed a different regional distribution. During the current 5 years, 7 prefectures showed statistically increasing trends in the incidence rates of legionellosis (Fig. 4c). The highest APC was seen in Tokushima (64.5%), followed by Wakayama (27.7%) and Gifu (24.7%).

### The reported numbers, crude, and AARs of death cases of legionellosis

**Figure 5:**
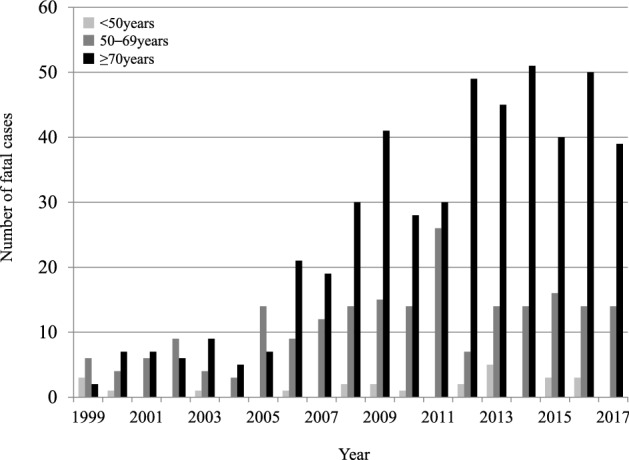
Trends in the number of fatal cases by age category for legionellosis in Japan, 1999–2017. The figure shows the trends in the number of fatal cases by age group and an increase in incidence especially in those aged over 70 years from 1999 to 2017.

During the study period, 725 fatal cases (569 men and 156 women) were reported in Japan. All the death cases were legionella pneumonia (A48.1), not including Pontiac fever (A48.2). Population aged ≥ 70 years accounted for 67% of the fatal cases; the number of death cases rose with age, with a peak at 75–79 years in men and 85–89 years in women (Fig. 2b). The number of deaths increased from 1999 to 2017 (53 cases overall; 41 in men and 12 in women). Both crude rate and AAR per 100,000 population in the last decade ranged between 0.03 and 0.05. Compared to the younger generations (< 50 years and 50–69 years), more deaths were observed in those aged ≥ 70 years (Fig. 5). Both in men and women, the number of deaths were very infrequent among those aged < 50 years. Compared to men, almost all the death cases in women were reported in those aged ≥ 70 years (Supplementary Figure S1).

**Figure 6:**
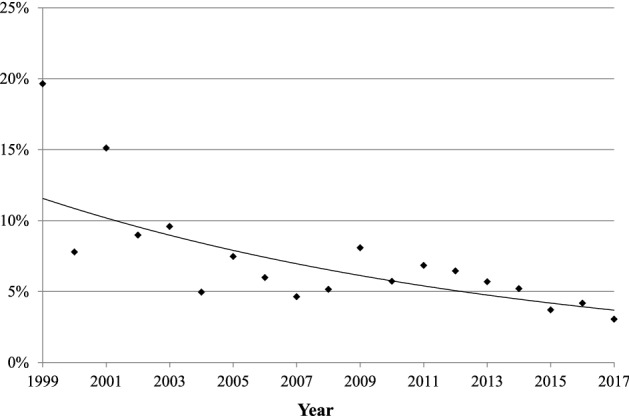
Trends in the estimated case fatality rate of legionellosis in Japan, 1999–2017. The annual estimated case fatality rate is plotted to show an approximate curve. The trend analysis also shows a decrease in the estimated mortality from 1999–2017. For details, see Table 2.

The trend in the estimated fatality rate of legionellosis is shown in Fig. 6. The overall estimated fatality rates decreased continuously at a rate of 5.9% (95% CI; − 8.1, − 3.5) annually from 1999 to 2017, without a trend change point. The declining trend was far significant in men (− 8.6%) than in women (− 5.2%) (Table 2).

## Discussion

We herein uncovered trends in the incidence and estimated fatality of legionellosis in Japan over nearly 2 decades by utilising a national database that is open to the public. Notably, we also analysed seasonality and regionality of the disease across the country; which has yet to be well described in the literature.

Similar to other countries^4–9^, the number of legionellosis incidence has increased continuously in Japan with 0.0004 to 1.37 reported cases per 100,000 population from 1999 to 2017 (Fig. 1). According to the NIID^16^, there was a sharp increase in the number of notifications after 2005 when the Legionella urinary antigen test was introduced as recommended in the clinical guidelines of the Japanese Respiratory Society. Internationally, the use of the urinary antigen test has been recommended for all patients with community-acquired pneumonia who require hospitalisation^17^. Thus, we speculate that the urinary antigen test was also promoted in Japanese clinical environments, for moderate or severe pneumonia cases, resulting in the increased numbers of appropriate diagnosis. The reported number of the disease then showed a plateau level for a few years, which again has trailed a rising trend until the end of the study period. This change can be explained by an availability of the loop-mediated isothermal amplification (LAMP) test in 2011, newly listed as an officially-approved test that is covered by the medical insurance. Focusing on sex, men accounted for the majority of the cases in both incidence and mortality, which has also been mentioned in previous literatures^2, 18^. This sex difference can be explained by the predisposing risk factors of the disease, including current/former smoking and heavy alcohol drinking^19^. As the analysis was based on aggregated publicly available data, we were unable to investigate underlying conditions in individuals with legionellosis.

As previously commented in a related article^5^, several factors can explain the increasing trend of legionellosis cases. The growing awareness among physicians has resulted in increases in the number of specimens submitted for the legionella-specific tests as described above. Actually, in Europe, it has been mentioned that improved surveillance was attributed to the continued increase of legionellosis incidence^20, 21^. Although nearly 90% cases of legionellosis in Japan has been diagnosed using the urinary Legionella antigen^16^, the detection rate based on the recently-available LAMP method is better than that^22^. Thus, the clinical incorporation of LAMP testing, starting from 2011, is also a key factor in the growing number of legionellosis in Japan. Additionally, the rise in the number of vulnerable people with risk factors for the disease, such as overlaps of underlying diseases, immunosuppressive drugs, and aging, can be attributed to the increasing trend of the legionellosis incidence^23^.

Of these causes, we consider age as an important factor. In this study, we discovered that legionellosis mainly involved the population aged ≥70 years; they represented approximately over 40% of all the reported cases (Fig. 2). A previous study also showed a higher number of cases in people aged >65 years, especially those with debilitating conditions^19^. In this rapidly-greying population, the clinical impact of the disease will continue to rise. The observed increasing trends of legionellosis in Japan are alarming to other countries as well, where aging is progressing.

Our study also suggested a seasonality of legionellosis incidence, that is more common in summer than winter (Fig. 3). Previous studies revealed that legionellosis appears more frequently during summer-autumn seasons rather than at other times of the year in the Northern Hemisphere^4, 9, 10^. This has possibly been accelerated by warmer and humid weather that we have been experiencing in recent years^5, 24^. In literature, an increase was reported in temperature and rainfall, as well as higher relative humidity, which have been associated with a higher risk of legionellosis^20, 25^. There is hardly a clear explanation, but the increased growth of *Legionella* bacteria in the warm environment is biologically plausible^26^. The on-going global warming, including in Japan^27^, may possibly explain the seasonality of legionellosis that may result in an increasing incidence of the disease in the future.

The regionality of legionellosis in Japan was also investigated in this study (Fig. 4). The incidence rates were highest in 3 prefectures in Hokuriku District (≥2.0 per 100,000 people). The trend analysis did not shown a recent increase in the incidence of legionellosis in these regions; however, there was a statistically significantly increase in the incidence of legionellosis in some other regions in Hokkaido and the middle-part of the country. There can be several confounding factors such as reporting bias, and we cannot provide a clear explanation for the regional differences. However, this is the first in the literature to uncover the regionality of the disease in Japan. The factors underlying these regional differences should be investigated in future research.

Our trend analysis suggested a decreasing trend in the estimated fatality of legionellosis in Japan, although the reported number of fatal cases has not been declining (Figs. 5 and 6). A case-series study also showed that the in-hospital case-fatality rate of legionellosis has decreased recently^19^. The decreasing trend in legionellosis can be attributed to the development of widely-available testing methods. In the past, having less resources to diagnose the disease, patients with legionellosis might not have been treated appropriately in its early phase. A retrospective cohort study reported that delayed commencement of appropriate therapy is associated with increased mortality^28^. Another study showed that difference in the sequence type of *Legionella pneumophila* was not associated with increased mortality, while delayed diagnosis by undetectable types led to delayed treatment and worsened prognosis, suggesting the importance of early diagnosis^29^. With the introduction of clinical laboratory diagnostic methods including bacterial culture, direct fluorescent antibody, and serologic testing, the case-fatality rate has decreased steadily since the mid-1980s to 1998^30^. Currently, with the availability of the urine antigen test and LAMP test, an increasing number of legionellosis is being diagnosed in Japan, contributing to reduced fatality. In addition, establishment of effective treatment for legionellosis (azithromycin and fluoroquinolone agents)^31, 32^ has also contributed to the declining fatal rates.

This study made a strong point of statistically analysing the recent trends in legionellosis incidence in Japan using the national database in about 2 decades. Through this study, we revealed an increasing trend in incidence, seasonality, regionality, and decreasing estimated fatality of legionellosis in Japan. Several limitations of the study should also be mentioned. First, under-reporting of the disease could have resulted in an underestimation of legionellosis. While misclassification of the disease could be minimal since the diagnosis of the disease is considered to be based on specific tests of urinary antigen and LAMP tests. The LAMP test used in Japan can detect 11 *Legionella* species including *L. pneumophila*^33^; but it does not detect all *Legionella* species. According to the Legionella Reference Centre in Japan, of 427 *Legionella* species isolated between 2008 and 2016, only 8 (1.8%) species other than *L. pneumophila* cause legionellosis^34^. Thus, we believe that the contribution of unidentified *Legionella* species to the incidence and mortality of legionellosis in Japan has decreased since the introduction of the LAMP test in 2011. Globally, the prevalent strains vary by region^35^, so the contribution of unidentified *Legionella* species may be higher in some other countries. Second, the case fatality rate was calculated by using the overall number of deaths and the total notification reports of the legionellosis cases. Appropriately, the case fatality rate should have been derived from individual data, not aggregated data. Thus, we would rather refer to this as an estimated case fatality rate. Third, the reliability of the death certificate data, from which legionellosis as an underlying cause of mortality was extracted, is uncertain; this is mainly due to fewer number of associated studies. As might be expected, there could have been under-reporting of cases. However, sensitivity, as well as the positive predictive value of death certificate data for the disease is reportedly adequate^36^, and thus, we believe that our trend analysis possibly reflects the entire picture of legionellosis in Japan. Fourth, due to the unavailability of data, our study lacked age information on the incidence of legionellosis among those aged ≥ 70 years. Finally, due to the absence of clinical data, we could not determine whether legionellosis was directly associated with the outcome in the reported patients. Despite these limitations, this study provides prominent findings to deeply understand the prevalence and characteristics of legionellosis in Japan.

In summary, we conducted a trend analysis of legionellosis in Japan from 1999 to 2017. The incident cases increased continuously during the study period; however, the estimated case fatality rates favourably declined. With increasing awareness among the physicians, further improvement in the diagnostic test, the growing number of ageing people, and the warming temperature, the incidence of legionellosis will be on an increasing trend. Our study informs the current trend and characteristics of the disease in Japan, providing prominent findings for better policy-making to further decrease the incidence and fatality.

## Methods

### Data source

This was a 19-year retrospective observational study including data from 1999 to 2017. The authors assert that all procedures contributing to this work comply with the ethical standards of the relevant national and institutional committees on human experimentation and with the Helsinki Declaration of 1975, as revised in 2008. Ethics approval was obtained from the institutional review board of the Okayama University Hospital (No. 1910–009).

The requirement for informed consent was waived because the study was a retrospective analysis of routinely collected data. The requirement for informed consent was waived by the ethics committee/IRB of Okayama University Graduate School of Medicine, Dentistry and Pharmaceutical Sciences and Okayama University Hospital because the study was a retrospective analysis of routinely collected data.

Data for the new cases of legionellosis were obtained from the Infectious Diseases Weekly Report, Japan^37^. Since 1999 April, clinical data on patients with stipulated diseases have been accumulated at the NIID, based on the Act on the Prevention of Infectious Diseases and Medical Care for Patients with Infectious Diseases (the Infectious Diseases Control Law). Legionellosis is one of the notifiable diseases, classified under Category IV Infectious Diseases that should be immediately reported after diagnosis^38^. Thus, when patients with the disease are diagnosed, medical practitioners are responsible for informing the public health centres, the reported data are then summarised by the National Epidemiologic Surveillance of Infectious Disease and made available to the public, in an online website^37^. Accordingly, cases diagnosed as legionellosis are well reported throughout the entire country. In this study, the incidence data of legionellosis included both for Legionella pneumonia and Pontiac fever. To compare the seasonality of the onset of legionellosis in Japan, we divided a whole year into four seasons: winter (1st to 13th week); spring (14th to 26th week); summer (27th to 39th week); and autumn (40th to 52nd; or 53rd week). To avoid reporting bias when calculating seasonality, we excluded the year when the total number of the annual incidence was < 500 cases. To compare the regional incidence and increasing trend of legionellosis in Japan, we calculated the mean rate per 100,000 population and the APC for each region. We utilised a geography software (https://www.sinfonica.or.jp/kanko/estrela/refer/s35/index.html) to draw differences in notification rates and its increasing trends among prefectures^39^.

Data on the number of deaths due to legionellosis were collected from the vital statistics, provided by the Japanese Ministry of Health, Labour, and Welfare^40^. Definitions of legionellosis deaths were according to the International Statistical Classification of Diseases and Related Health Problems coding system (tenth edition) as previously reported; legionella pneumonia (A48.1) and Pontiac fever (A48.2)^18, 41^. We categorised age as < 50 years, 50–69 years, and ≥ 70 years. To improve comparability, we adopted the direct standardisation method and calculated the AARs of incidence and mortality, based on the Japanese population in 1999, the first year of the study period. For the incidence of those aged 0 to 70 years, we applied 5-yearly age groups for the standardisation. For those aged ≥ 70 years, the 5-yearly age group data was unavailable; thus, we treated the data for those aged ≥ 70 years as a combined number. We calculated the estimated fatality rate by dividing the number of fatal cases by the number of the reported incident cases in each year.

### Statistical analyses and data processing

We calculated the incidence and mortality crude and AARs of legionellosis. We used the 1999 population as the standard population for calculating the age-adjusted incidence rates. To estimate the trends in the AAR, the Joinpoint regression model was applied by sex and age using the Joinpoint Regression Program, version 4.5.0.1, June 2017 (Statistical Research and Applications Branch, National Cancer Institute)^42^. The analysis identified the year when the significant trend changes occurred and estimated the magnitude of the increase or decrease. Each joinpoint showed a statistically significant change in trend, and APC was calculated for each of those trends by means of the generalised linear models assuming a Poisson distribution^43^. Also, the AAPC in the entire period was estimated. For the comparison of the incidence among the 4 seasons, we applied the Kruskal–Wallis analysis using the EZR software, a graphic user interface for the R 3.5.2 software (The R Foundation for Statistical Computing, Vienna, Austria)^44^. All the processes of data collections and calculations were performed independently and in duplicate by 2 distinct researchers (SF and YO) to ensure accuracy of the analysis. All reported P values < 0.05 were considered statistically significant.

## Supplementary Information

Supplementary Information.

## Data Availability

The datasets generated and analysed during the current study are available from the corresponding author on reasonable request.
