# Supplementary material for: Trends in the incidence and mortality of legionellosis in Japan: a nationwide observational study, 1999–2017
Source: Sci Rep. 2021 Mar 31;11:7246. doi: 10.1038/s41598-021-86431-8 (PMC8012643; doi:10.1038/s41598-021-86431-8)

Title:

Trends in the Incidence and Mortality of Legionellosis in Japan: A Nationwide Observational Study, 1999–2017

Authors and affiliations:

Shinnosuke Fukushima, MD [1)], Hideharu Hagiya, MD, Ph.D. [1)]*, Yuki Otsuka, MD [1)], Toshihiro Koyama, Ph.D. [2)], Fumio Otsuka, MD, Ph.D. [1)]

1) Department of General Medicine, Okayama University Graduate School of Medicine, Dentistry and Pharmaceutical Sciences, 2-5-1 Shikata-cho, Kitaku, Okayama, 700-8558
2) Department of Pharmaceutical Biomedicine, Graduate School of Medicine, Dentistry, and Pharmaceutical Sciences, Okayama University, 1-1-1 Tsushima-Naka, Kita-ku, Okayama, 700-8530, Japan

***Corresponding Author:**

Hideharu Hagiya, MD, PhD

Department of General Medicine, Okayama University Graduate School of Medicine, Dentistry and Pharmaceutical Sciences, 2-5-1 Shikata-cho, Kitaku, Okayama, 700-8558

TEL: +81-86-235-7342; FAX: +81-86-235-7345

E-mail: hagiya@okayama-u.ac.jp

Supplementary Table S1. Mean incidence rate (per 100,000 population) and annual percentage change of legionellosis across 47 prefectures in Japan, 1999–2017

	Incidence	APC [95% CI]
Hokkaido*	0.69	10.0 [0.3, 20.7]
Aomori	0.48	22.0 [-13.8, 72.7]
Iwate	1.33	11.8 [-27.7, 72.7]
Miyagi	1.81	-2.7 [-36.9, 49.9]
Akita	1.41	16.5 [-48.5 163.5]
Yamagata	1.37	-1.2 [-15.3 15.4]
Fukushima	1.21	18.3 [-7.3, 51.0]
Ibaraki	1.55	9.9 [-16.8, 45.2]
Tochigi	1.65	10.9 [-17.2, 48.4]
Gunma	1.78	18.8 [-15.8, 67.6]
Saitama*	1.03	21.6 [0.3, 47.4]
Chiba	1.06	3.2 [-8.4, 16.3]
Tokyo	0.95	15.2 [-1.4, 34.7]
Kanagawa	1.24	4.7 [-25.8, 47.7]
Niigata	1.62	-0.5 [-14.1, 15.2]
Toyama	3.49	1.1 [-10.9, 14.8]
Ishikawa	2.81	-1.3 [-25.4, 30.6]
Fukui	2.04	23.2 [-26.7, 106.8]
Yamanashi	0.94	13.6 [-30.6, 86.0]
Nagano*	1.67	11.1 [4.8, 17.9]
Gifu*	1.30	24.7 [2.0, 52.3]
Shizuoka*	1.22	16.6 [5.4, 28.9]
Aichi	1.13	13.8 [-2.1, 32.3]
Mie	1.17	9.9 [-3.5, 25.1]
Shiga	1.57	7.9 [-20.0, 45.5]
Kyoto	1.02	11.3 [-21.6, 57.8]
Osaka	0.85	7.1 [-0.7, 15.6]
Hyogo*	1.07	14.7 [5.4, 24.9]

Nara	0.87	10.9 [-16.7, 47.5]
Wakayama	0.92	27.7 [-3.6, 69.2]
Tottori	1.08	-0.4 [-26.0, 34.1]
Shimane	0.81	-7.4 [-39.7, 42.0]
Okayama	1.46	2.3 [-10.0, 16.2]
Hiroshima	1.73	40.1 [-5.7, 108.2]
Yamaguchi	0.89	15.9 [-1.7, 36.6]
Tokushima*	0.93	64.5 [11.5, 142.7]
Kagawa	0.94	2.1 [-47.5, 98.6]
Ehime	0.93	15.2 [-25.9, 79.0]
Kochi	0.52	21.4 [-9.8, 63.6]
Fukuoka	0.82	11.5 [-1.1 25.6]
Saga	0.46	-15.9 [-56.5, 62.8]
Nagasaki	0.79	18.6 [-12.5,60.8]
Kumamoto	1.40	21.3 [-20.6, 85.3]
Oita	1.25	1.7 [-21.1, 31.1]
Miyazaki	0.65	-6.0 [-47.6, 68.5]
Kagoshima	0.53	21.7 [-48.4, 186.7]
Okinawa	1.13	0.4 [-25.9, 35.9]

*Prefecture with a significantly increasing trend.

CI, confidence interval.

Supplementary Figure S1. Trends in the number and the fatal cases by sex and age category, 1999–2017

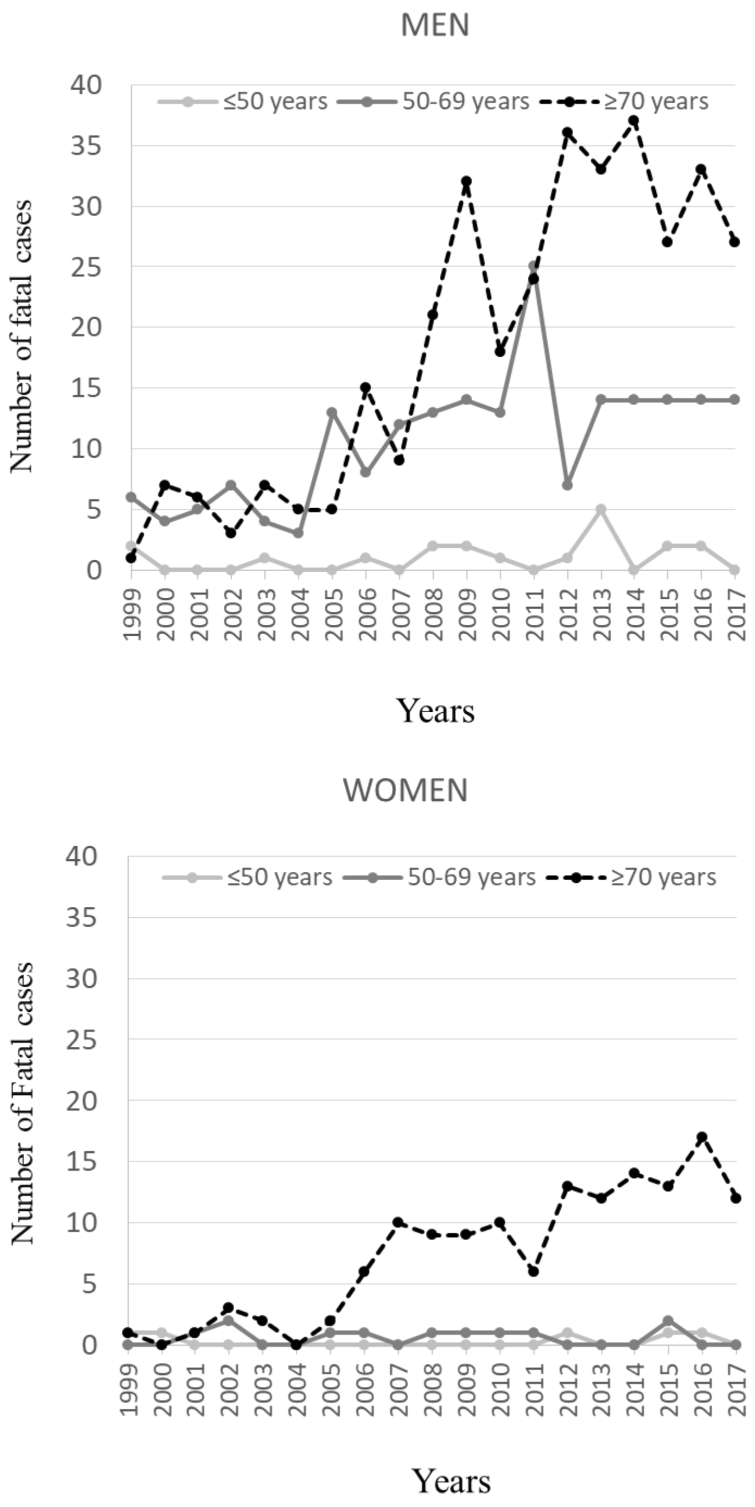

Supplement: Supplementary file 1 — Supplementary Information. [file 41598_2021_86431_MOESM1_ESM.pdf]
